# Central nervous system zoning: How brain barriers establish subdivisions for CNS immune privilege and immune surveillance

**DOI:** 10.1111/joim.13469

**Published:** 2022-03-14

**Authors:** Steven T. Proulx, Britta Engelhardt

**Affiliations:** ^1^ Theodor Kocher Institute University of Bern Bern Switzerland

**Keywords:** blood–brain barrier, blood–cerebrospinal fluid barrier, glia limitans, immune surveillance, lymphatic system

## Abstract

The central nervous system (CNS) coordinates all our body functions. Neurons in the CNS parenchyma achieve this computational task by high speed communication via electrical and chemical signals and thus rely on a strictly regulated homeostatic environment, which does not tolerate uncontrolled entry of blood components including immune cells. The CNS thus has a unique relationship with the immune system known as CNS immune privilege. Previously ascribed to the presence of blood–brain barriers and the lack of lymphatic vessels in the CNS parenchyma prohibiting, respectively, efferent and afferent connections with the peripheral immune system, it is now appreciated that CNS immune surveillance is ensured by cellular and acellular brain barriers that limit immune cell and mediator accessibility to specific compartments at the borders of the CNS. CNS immune privilege is established by a brain barriers anatomy resembling the architecture of a medieval castle surrounded by two walls bordering a castle moat. Built for protection and defense this two‐walled rampart at the outer perimeter of the CNS parenchyma allows for accommodation of different immune cell subsets and efficient monitoring of potential danger signals derived from inside or outside of the CNS parenchyma. It enables effective mounting of immune responses within the subarachnoid or perivascular spaces, while leaving the CNS parenchyma relatively undisturbed. In this study, we propose that CNS immune privilege rests on the proper function of the brain barriers, which allow for CNS immune surveillance but prohibit activation of immune responses from the CNS parenchyma unless it is directly injured.

## Graphical abstract

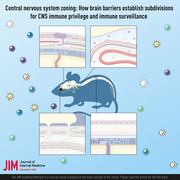

## Immune surveillance: General principles of innate and adaptive immunity

The immune system has evolved to protect the body from attacks from microbial pathogens and trauma and is thus ultimately tuned to ensure host survival in a hostile environment [1, 2]. The most exposed sites for such encounters are the outer and inner body surfaces including the skin, the gut, and the respiratory tract. The epithelial linings at these interfaces establish a physical barrier and are fortified by site‐specific immune defense mechanisms provided by the accumulation of innate and adaptive immune cells immediately behind the epithelial borders (summarized in [3]). Encounter with microbes or physical and chemical trauma leads to rapid stereotypic activation of tissue resident innate immune mechanisms based on recognition of pattern‐associated molecular patterns (PAMPs) on microbes or damage‐associated molecular patterns (DAMPs) from injured host cells by tissue resident macrophages. Next, the local release of inflammatory cytokines and chemotactic factors induces the expression of trafficking molecules on blood vascular endothelial cells, which initiates the recruitment of circulating neutrophils and myelomonocytic cells to the affected site [4]. Together with the tissue resident macrophages, the infiltrating neutrophils and monocyte‐derived macrophages kill and phagocytose the microbes, digest injured cells of the host and, in most cases, eventually initiate resolution of the inflammatory response. This innate immune response also includes activation of tissue resident dendritic cells (DCs), which are professional antigen‐presenting cells (APCs) that take up and process antigens and carry them via the afferent lymphatic vessels to the tissue‐draining lymph nodes, where the antigen is presented on major histocompatibility (MHC) molecules to naïve T cells, thus enhancing the likeliness that the appropriate T‐cell encounters its cognate antigen. T cells that recognize their cognate antigen on the DC with their T‐cell receptor will proliferate and differentiate into effector T cells. B cells can recognize soluble antigens with their B‐cell receptor, which triggers antigen internalization and presentation of these antigens to helper T cells in the lymph node, which, as their name suggests, provide “help” for maximal B‐cell activation and proliferation resulting in the differentiation into antibody‐producing plasma cells.

During their activation naïve lymphocytes are imprinted with trafficking programs (expression of a combination of adhesion and chemoattractant receptors) that—once they have via the efferent lymphatics reached the bloodstream—ensure their migration back to the tissue drained by the respective lymph node and thus direct site‐specific adaptive immune responses. For example, in gut and skin draining lymph nodes DCs have been shown to play an important role in this process by processing food‐derived vitamin A and ultraviolet‐induced vitamin D_3_, respectively, to imprint gut homing and skin homing trafficking programs as well as site‐specific effector functions in naïve lymphocytes (summarized in [5]). With their respective effector functions, that is, cytokine production, killing of infected tissue cells, and antibody production, the adaptive immune cells ensure elimination of the injurious agent and reconstitution of tissue function.

In contrast to the innate immune system, the adaptive immune system has the ability to establish a memory of previous antigen encounters and thus provides accelerated immune responses in case of a further encounter with the same antigen. Site‐specific cellular immune memory is hereby realized by tissue‐resident memory T (T_RM_) cells expressing the T‐cell activation marker CD69 and the αE‐integrin subunit CD103, which when associated with the β7‐integrin subunit forms αEβ7‐integrin, a ligand for E‐cadherin, the adhesion molecule forming epithelial adherens junctions [6]. αEβ7‐integrin may thus serve as a retention molecule for barrier‐associated T_RM_ cells. Systemic cellular immune memory is realized by central memory T (T_CM_) cells that re‐express L‐selectin and the chemokine receptor CCR7—the trafficking program of naïve lymphocytes—allowing T_CM_ cells to recirculate through lymph nodes throughout the entire body, where they can then provide accelerated immune responses to their cognate antigen even if encountered at a different anatomical entry site (summarized in [7]). Cellular immune memory is accompanied by the humoral immune response established by tissue resident and circulating memory B cells, in addition to plasmablasts that either specifically home back to the mucosal barriers, where they differentiate into plasma cells producing high amounts of antibodies of the immunoglobulin (Ig) A class, or home back to the bone marrow, where they differentiate into plasma cells releasing high affinity antibodies into the bloodstream. Immune surveillance of a given tissue thus relies on drainage by lymphatic vessels to transport antigens and antigen‐presenting DCs to the draining lymph nodes, as well as on blood vessels to allow for efficient immune cell entry into the respective tissues.

## Toward understanding CNS immune privilege and CNS immune surveillance

The anatomical location of the CNS, that is the brain, the neural part of the eye, and the spinal cord, within the skull and vertebral column provides robust protection toward the outside of the body. Unless there is a penetrating injury, foreign material or pathogens are thus quite unlikely to directly reach the CNS unless they have escaped the specific innate and adaptive immune defense mechanisms at the epithelial surfaces of the skin, the gut, and respiratory tract. In this case, pathogens could reach the CNS via the bloodstream or potential routes along with cranial (e.g., from the nasal mucosa) and spinal nerves (e.g., from the gut). However, the CNS resides behind blood–brain barriers (BBBs) that restrict pathogen and immune cell entry from the periphery into the CNS parenchyma and also lacks lymphatic vessels. The CNS must thus have a unique relationship with the immune system that differs from that of peripheral organs.

Indeed, experiments performed originally by Shirai 100 years ago [8] and followed up and extended by Medawar and others [9] showed that foreign tissues when grafted to peripheral sites like the skin were readily rejected, but when grafted into the brain parenchyma or anterior chamber of the eye survived for prolonged durations. These organs, in which experimentally implanted tissue grafts are incapable of provoking immunity leading to graft rejection, were subsequently referred to as “immune privileged organs” [10]. Subsequent observations showed that CNS immune privilege also extends to innate immune responses. Neither injection of bacterial products and thus exposure to PAMPs [11] nor experimental induction of cell death and thus exposure to DAMPs [12, 13] within the CNS parenchyma elicits a rapid infiltration of myelomonocytic cells into the tissue as observed during a stereotypic response to such stimuli in peripheral organs [4]. Based on these observations, the idea emerged that antigens contained within the CNS could not be seen by the immune system as lack of conventional lymphatic vessels in the CNS parenchyma would prohibit the afferent arm of the immune system, that is, drainage of CNS antigens into peripheral lymphatic tissues, while the endothelial BBB would inhibit the efferent arm of the immune system by blocking immune cell entry into the CNS (summarized in [14, 15]).

Ironically, this concept of “CNS immune ignorance” literally “ignored” the additional observations of Medawar [9], which showed that foreign tissue grafts transplanted into the brain parenchyma of animals that had previously rejected a tissue graft of the same donor in the skin were readily destroyed. Medawar thus concluded already in 1948 that “skin homografts transplanted to the brain submit to but do not elicit an immune state.” Intriguingly, tissue grafts transplanted into the cerebral ventricles instead of within the brain parenchyma were in following studies found to be rejected [16, 17], underscoring that within the CNS there are compartments that differ with respect to their relationship with the immune system. Subsequent observations showing that cerebrospinal fluid (CSF) drains into cervical lymph nodes [18] and that activated circulating T cells can cross the BBB even in the absence of neuroinflammation (summarized in [19]), finally called for revisiting the concept of CNS immune privilege. We have thus proposed that comparable to the architecture of medieval castles that were designed for protection and defense, the CNS of vertebrates has evolved a similar architecture of brain barriers that allow for different levels of immune surveillance [20]. In this concept, the CNS parenchyma is immune‐privileged allowing it to prioritize proper function of neurons over eliciting an immune response, while the CNS ventricular spaces and border compartments (subarachnoid and perivascular spaces) akin to the castle moat are dedicated to CNS immunity and thus lack full CNS immune privilege [21].

## Role of the brain barriers in CNS immune surveillance

**Fig. 1:**
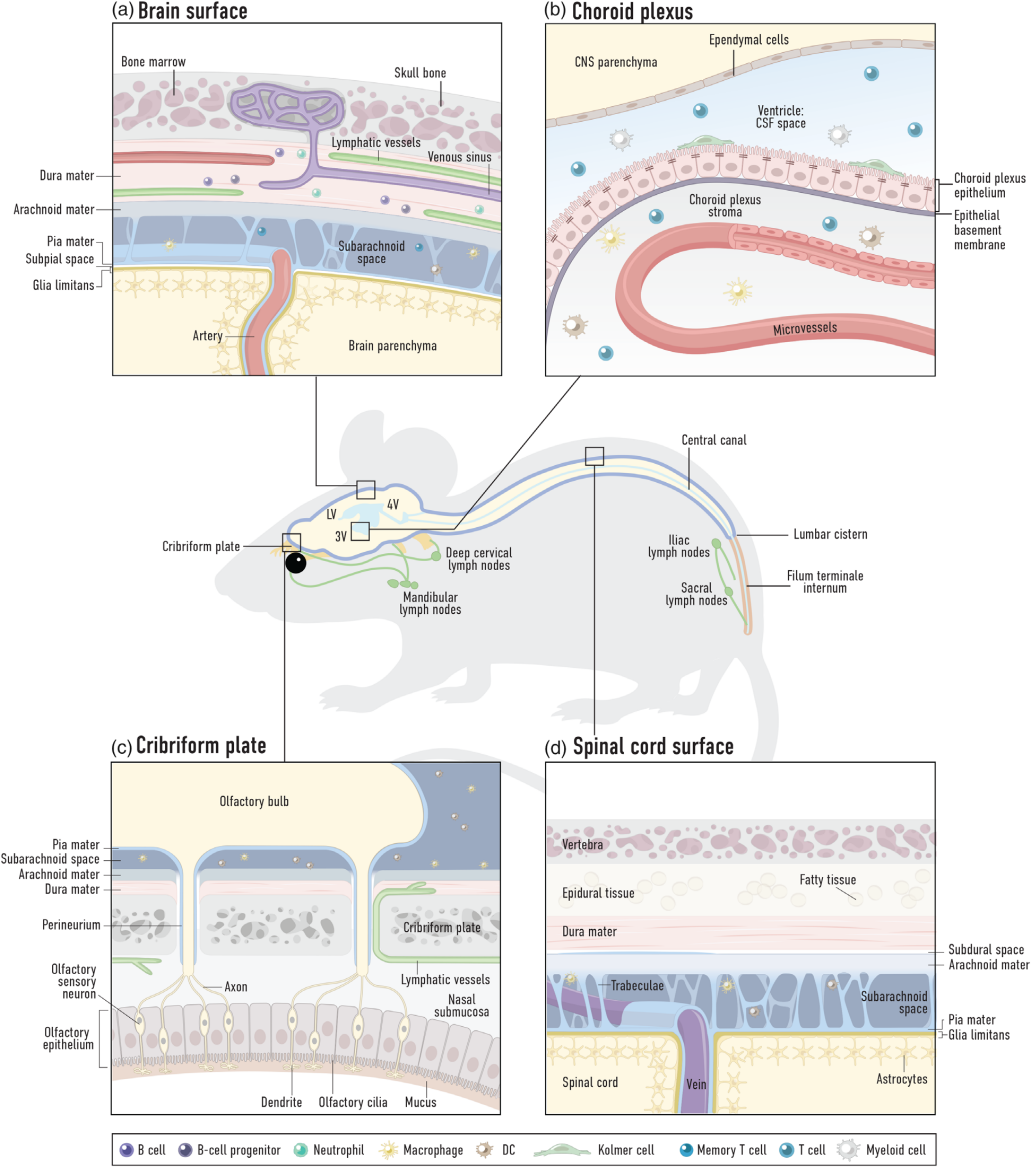
Overview of the brain barriers at select regions of the central nervous system (CNS). The mouse schematic displays the ventricular system (blue), comprised of two lateral ventricles (LV), the third ventricle (3V), the fourth ventricle (4V) and the central canal of the spine, and several known egress sites for cerebrospinal fluid (CSF) along the spinal and cranial nerves (yellow) to lymphatic vessels and draining lymph nodes (green). (a) Barriers at the dorsal surface of the brain cortex. The brain parenchyma is wrapped by the glia limitans, comprised of astrocyte end‐feet and an associated parenchymal basement membrane. Overlaying this is the pia mater meningeal layer, which along with the arachnoid mater encloses the subarachnoid space containing CSF and innate and adaptive immune cells performing CNS immune surveillance. The outermost layer of the meninges, the dura mater, contains venous sinuses draining the brain tissue, as well as its own blood vascular network (that does not form a blood–brain barrier) and dural lymphatic vessels. Peripheral immune cells thus have regular access to the dura mater. Channels surrounding blood vessels between the dura mater and bone marrow cavities may allow communication between these two compartments. (b) Blood–CSF barrier at the choroid plexus. The choroid plexus stroma is fed by a blood vessel network not endowed with barrier properties and harbors innate and adaptive immune cells. The barrier between this tissue and the CSF in the ventricles is formed by a layer of choroid plexus epithelial cells and an associated basement membrane. The CSF harbors low numbers of mainly adaptive immune cells performing CNS immune surveillance, as well as Kolmer (epiplexus) cells. The ventricle is lined by ependymal cells, which allows exchange of solutes between the CSF and the brain parenchyma. (c) CSF efflux routes at the cribriform plate. Olfactory nerve bundles extending from the olfactory bulbs project through foramina of the cribriform plate of the ethmoid bone to terminate at the epithelial layer of the nasal turbinates. The pia mater covering the olfactory bulbs blends into the perineurium surrounding the nerve bundles. At this region, the arachnoid layer is disrupted allowing passage of fluid, solutes (and possibly cells) out of the subarachnoid space. Lymphatic vessels, found either within the nasal mucosa or passing through the foramina along with the olfactory nerve bundles to the CNS side of the cribriform plate, are responsible for uptake and transport to the periphery. (d) Barriers at the surface of the dorsal spinal cord. The spinal cord parenchyma is wrapped by the glia limitans, comprised of astrocyte end‐feet and an associated parenchymal basement membrane. Veins exiting the spine are ensheathed by pia mater. The subarachnoid space lies between the pia mater and arachnoid mater and is traversed by trabeculae. It harbors innate and adaptive immune cells performing CNS immune surveillance. The spinal cord dura (unlike cranial dura) is associated with a subdural space and epidural tissue.

The brain barriers simultaneously maintain homeostasis of the CNS parenchyma while enabling immune surveillance at the CNS borders (Fig. 1). The outer borders of the brain and spinal cord are ensheathed by three meningeal layers, namely, the dura mater, the arachnoid mater, and the pia mater [22]. The arachnoid mater hereby establishes the blood–arachnoid barrier, which is a bona fide blood–cerebrospinal fluid barrier (BCSFB), between the outer dura mater lacking a BBB and the inner CSF‐filled subarachnoid space [23]. Another BCSFB is found in the choroid plexuses that produce the CSF and extend from the ventricular walls into the brain ventricles. The choroid plexus stroma harbors fenestrated blood vessels and are separated from the ventricular space by choroid plexus epithelial cells establishing another BCSFB [24]. The bona fide BBB is established by CNS parenchymal microvascular endothelial cells [25]. Finally, the glia limitans is established by astrocytes that ensheath the entire CNS parenchyma with their end‐feet and support a parenchymal basement membrane [26].

Complex tight junctions between the endothelial cells of the BBB and between the choroid plexus epithelial cells and arachnoid fibroblasts of the BCSFBs inhibit paracellular diffusion of solutes, while polarized expression of transporters and efflux pumps at these cellular barriers ensure transport of nutrients into and the rapid export of toxic metabolites out of the CNS [21, 27‐29]. In ancestral vertebrates, the BBB was formed by glial cells, and evolutionary pressure led to the development of the endothelial BBB [30]. In modern vertebrates, the glial barrier has remained as the *glia limitans* and establishes a second barrier “behind” the BBB and BCSFB, which in the healthy CNS prohibits immune cell entry into the CNS parenchyma (summarized in [26, 31]). The two‐walled defense system around the CNS parenchyma is thus established by the BBB and the BCSFBs toward the periphery and the *glia limitans* toward the CNS parenchyma, allowing for CNS immune surveillance in the CSF‐communicating perivascular and CSF‐filled subarachnoid spaces. In keeping with the analogy of medieval castle architecture, an efficient defense system requires castle guards. To this end, the CNS harbors two types of tissue‐resident macrophages that originate from the yolk sac, invade during embryonic development, and self‐renew in their respective niches throughout adulthood (summarized in [32]). While microglial cells are localized in the CNS parenchyma where they regulate synaptic pruning and thus neuronal cross‐talk [33], CNS border‐associated macrophages are found at the apical CSF‐facing side of the choroid plexus (known as epiplexus macrophages or Kolmer cells) and in the perivascular and subarachnoid spaces. Thus, by location the latter cells are assigned the task of the castle moat sentries that monitor the CSF and are ready to react to any PAMPs or DAMPs.

There are no tissue‐resident T cells or B cells in the CNS parenchyma of healthy young adults, but there are low numbers of immune cells found in the CSF and these are dominated by T cells with small numbers of monocyte‐derived dendritic cells (mDCs) and CSF‐specific monocytes combining transcriptional signatures of monocytes, CNS border‐associated macrophages, and microglial cells [34, 35, 36]. Single cell RNA sequencing has shown that CSF T cells are mainly composed of memory CD4 and CD8 T cells that share characteristics with tissue‐resident T cells as well as regulatory T cells [34, 36]. In contrast, naïve T cells and B cells are largely excluded from the CSF [36]. Thus, although low in numbers, the CSF spaces do contain a full repertoire of cells of the innate and adaptive immune system ensuring CNS immune surveillance from these border locations.

As if this immune protection was not enough, the CNS has recently been referred to as immune‐fortified at its outer borders [37], where the dura mater—the outermost layer of the meninges ensheathing the brain and spinal cord—as well as the choroid plexus stroma are both rich in immune cells including macrophages, monocytes, neutrophils, natural killer cells, DCs, as well as T and B lymphocytes [33, 38–40]. The dura mater and the choroid plexus stroma lack a vascular BBB and are thus readily accessible for blood components including circulating immune cells. In fact, two‐photon in vivo imaging has allowed visualization of choroid plexus and dural macrophages and has shown that they continuously extend cellular processes to probe their surroundings preferentially in perivascular locations, thus ensuring to catch any danger arriving via the bloodstream [38, 41]. Immune cells localized in the dura mater and the choroid plexus stroma are thus ideally positioned to fortify the BCSFBs right outside of the CNS tissue proper.

In addition to blood‐derived immune cells, the dura mater may have access to immune cells directly originating from the bone marrow in the adjacent skull or vertebrae of the spine [39, 40]. These bone marrow‐derived cells were suggested to reach the dura mater by crawling along the outside of venules inside small bony channels [42]. Transcription profiling and phenotyping of the dura mater immune cells implied that bone marrow‐derived immune cells are rather programmed to maintain CNS health, whereas those arriving from the blood rather showed transcriptional profiles characteristic for immune cells ready to fight potential infections [39, 40]. It is furthermore very intriguing that under steady state conditions the dura mater harbors neutrophils [40, 43], a cell population of the innate immune system that usually circulates in the blood and is only recruited into tissues in response to acute injury. It is tempting to speculate that these neutrophils differ in their function from those in the circulation. It has been suggested that they may be the source of neutrophils accumulating in subarachnoid and perivascular spaces under pathological conditions such as ischemic stroke where therapeutic inhibition of neutrophil recruitment from the blood has failed [42, 44]. In this scenario, dura mater‐derived neutrophils would need to breach or circumvent the arachnoid barrier through mechanisms which remain to be shown. The dura mater was furthermore observed to contain B lineage progenitors that are typically not found outside of the bone marrow [39, 45]. While the presumptive skull bone marrow origin of dura mater B cells was not confirmed in the second study [45] both studies identify the dura mater as an unexpected site for B‐cell development and residence, which could provide CNS tailor‐made B‐cell responses including antigen presentation and antibody production at this outer brain barrier.

## Role of the brain barriers in regulating efferent pathways of CNS immunity

The vast majority of studies addressing the anatomical routes as well as cellular and molecular mechanisms of immune cell migration into the CNS have been performed in the context of experimental autoimmune encephalomyelitis (EAE), an animal model for multiple sclerosis (MS) [46]. By the nature of this model, which is a CD4 T‐cell‐mediated autoimmune neuroinflammatory disease, these studies have largely focused on encephalitogenic CD4 T‐cell subsets, namely, interleukin (IL)‐17 producing Th17 cells, and γ‐interferon producing Th1 cells, and provided robust evidence for three potential routes of CD4 T‐cell entry into the CNS [47]. Elegant intravital imaging studies have shown that these activated T cells can either cross subarachnoid or subpial veins to reach the subarachnoid space in the brain or spinal cord [48, 49] or migrate across subpial post‐capillary venules to reach perivascular spaces [50]. In addition, preferentially Th17 cells can reach leptomeningeal spaces of the brain using the choroid plexus as a CNS entry site [51]. Intravital microscopy studies have furthermore allowed for direct observation of the concept that an activated state rather than antigen‐specificity is required for T cells to cross subarachnoid or subpial venules [49], supporting the notion that a peripheral immune response mounted to an external insult will release activated T cells into the circulation that can reach the CNS.

Unless they recognize their cognate antigen on perivascular or leptomeningeal APCs—the “castle moat sentries”—these activated T cells will, however, not cross the glia limitans [52, 53] and instead either undergo apoptosis, differentiate into central memory T cells or tissue‐resident T cells that remain in the CSF [34, 36] or potentially leave the CNS by CSF‐drainage pathways. In contrast, local recognition of their cognate antigen on APCs (border‐associated macrophages or DCs) will lead to reactivation of these T cells in the subarachnoid or perivascular spaces and their subsequent crossing of the glia limitans into the CNS parenchyma [52, 54]. It is at this stage, when immune cells cross the glia limitans, that clinical disease in EAE starts [55]. The observations in the context of the EAE model underscore that immune cell penetration of the inner wall of the castle moat and their infiltration into the CNS parenchyma is not compatible with proper CNS homeostasis.

A study following the trafficking of fluorescently or radioactively labeled lymphocytes collected from the efferent lymph in sheep has questioned the requirement of recent activation for lymphocytes to reach the CSF [56]. This recirculating pool of lymphocytes was observed to reach the CSF from the bloodstream and CNS‐draining lymph nodes from the CSF. As already at that time the choroid plexus was proposed as alternative immune cell CNS entry site [57], the authors speculated that the CSF is patrolled by the recirculating pool of lymphocytes, which may access the CSF space via the choroid plexus [56] and leave the CNS via the CSF‐draining pathways reaching the cervical lymph nodes (as previously observed by others [58]) through pathways yet to be defined. Recent single cell transcriptome profiling comparing blood and CSF immune cells in humans showed, however, that although T cells display a phenotypic continuum between the blood and CSF, CSF T cells are largely distinct from those in the blood of the same individual [34, 36]. These previous studies performed in sheep could not distinguish between individual immune cell subsets in the circulating lymphocyte pool, which in sheep (which are not typically kept under specific pathogen‐free conditions) may also contain peripherally activated T cells. Thus, future studies should address if there is a subset of T cells that do indeed recirculate between the periphery and the CSF, while other T cells upon reaching the CSF will differentiate into CSF‐resident memory T cells ensuring immunological memory in this border compartment of the CNS.

The migration of T cells across the endothelial brain barriers is a multistep process regulated by the sequential interaction of different signaling and adhesion molecules on the CNS endothelium and the T cells and takes place at the level of subarachnoid and subpial venules or parenchymal post‐capillary venules [46]. Accounting for the unique barrier properties of the CNS microvascular endothelial cells, T‐cell migration across the endothelial brain barriers is characterized by unique adaptations. In contrast to venular endothelial cells in peripheral vascular beds, BBB endothelial cells lack constitutive expression of P‐selectin and the atypical chemokine receptor 1 (ACKR1), which mediate rolling and arrest/diapedesis of immune cells, respectively [59, 60, 61, 62]. Thus, in the absence of neuroinflammation, α4β1‐integrins play a predominant role in T‐cell entry into the CNS by mediating initial T‐cell capture and subsequent Gαi‐signaling‐dependent adhesion strengthening on the CNS endothelium by engaging endothelial VCAM‐1 [50]. Endothelial ICAM‐1 and ICAM‐2 next mediate T‐cell polarization and subsequent crawling over extended distances against the direction of blood flow on the surface of the CNS endothelium in search of tricellular junctions, which provide rare sites permissive for diapedesis [46, 54, 63]. Interestingly, the molecular makeup of BBB tricellular junctions with localized expression of tricellulin and angulin‐1 is different from that of tricellular junctions of peripheral endothelial cells [64], suggesting that BBB tricellular junctions provide a unique scaffold for T‐cell diapedesis probably by allowing the T cells to sense chemotactic factors like chemokines across the BBB [63]. During neuroinflammation, when barrier properties including junctional integrity of the BBB are disturbed and additional adhesion and trafficking molecules are induced on the BBB [65], an increase in transcellular T‐cell diapedesis via pores through the brain microvascular endothelial cells can be observed [66, 67]. Thus, the unique junctional complexes of the endothelial BBB seem to play an important role in controlling immune cell entry into the CNS during immune surveillance. The precise molecular mechanisms involved in this process remain to be investigated.

Studies in the context of EAE have furthermore suggested that while Th1 cells preferentially infiltrate the spinal cord using α4β1‐integrins [68, 69], Th17 cells preferentially enter the brain and require LFA‐1 but not α4‐integrins [70]. In fact, Th17 cells expressing the chemokine receptor CCR6 have been shown to reach the subarachnoid space via the choroid plexus where the choroid plexus epithelial cells from the BCSFB express the CCR6 chemokine ligand CCL20 [51]. It is generally assumed that Th17 cells leave the choroid plexus stroma by crossing the BCSFB into the ventricular CSF from where they reach the subarachnoid space. This is supported by observations in vitro, in which higher fractions of Th17 cells as compared to other Th cell subsets cross an epithelial BCSFB [71]. Key adhesion molecules involved in immune cell trafficking, like ICAM‐1 and VCAM‐1 although exclusively expressed on the apical side of the choroid plexus epithelium, seem to contribute to the last step of transepithelial T‐cell migration [71]. We and others have, however, also considered that T cells may exit the choroid plexus stroma at the base of the choroid plexus where it folds out from the ventricular wall [72]. Brain barrier structures such as tanycytes that surround the circumventricular organs that also lack a BBB have not been described at the base of the choroid plexus. Thus, studies exploring the detailed neuroanatomy at the base of the choroid plexuses, where they fold out into the ventricles are urgently needed. One study pointed out a direct continuity of the basement membrane of the choroid plexus epithelial cells with the parenchymal basement membrane of the glia limitans [73], implying a direct continuity of the choroid plexus stroma with the subarachnoid space. In this scenario, immune cells could reach the subarachnoid space directly from the choroid plexus stroma without passing any endothelial or epithelial outer brain barrier. In our model of CNS immune privilege, the role of the outer castle wall controlling immune cell entry into the castle moat would thus be attributed to the choroid plexus stroma where choroid plexus fibroblasts may provide the chemotactic cues regulating immune cell retention in the choroid plexus stroma and their potential release for crawling along the glia limitans directly into the subarachnoid space. In addition, the choroid plexus stroma is very rich in tissue‐resident and blood‐derived immune cells including APCs, and thus fortifies CNS immune protection just outside of the BCSFB and possibly at the outer border of the subarachnoid space.

Recent studies have also suggested that immune cells localized in the dura mater may reach the CNS from this site [39, 40, 45]. This would imply that dural immune cells cross the BCSFB established by arachnoid barrier cells, which has not been studied to date. Alternatively, as dural immune cells are highly concentrated along the dural sinus [74], one can speculate that dural immune cells may crawl along with perivenular structures that breach the arachnoid barrier at this location.

Once they have reached the subarachnoid space, elegant in vivo imaging studies have shown that activated T cells crawl on leptomeningeal cells in a random Brownian walk using α4β1‐integrin and LFA‐1 and eventually encounter border‐associated macrophages or would be flushed away by the flow of the CSF [75]. Recognition of their cognate antigen in the subarachnoid space triggers T‐cell activation and proliferation and a local inflammatory milieu that will initiate the migration of these locally activated T cells across the glia limitans and disturbance of CNS function visible as clinical disease [52, 55].

Under physiological conditions, the glia limitans provides a barrier for the migrating immune cells, which in the absence of neuroinflammation do not enter the parenchyma [26]. Astrocyte end‐feet tightly ensheathing the CNS parenchyma and a parenchymal basement membrane composed of laminins 111 and 211 that are not involved in T‐cell binding may explain in part the molecular mechanism behind this protective function [26]. In fact, degradation of astrocyte end‐feet extracellular matrix receptors by matrix metalloproteinases (MMPs) during neuroinflammation correlates with immune cell crossing of the glia limitans [55]. At the same time, this MMP activity changes the chemokine milieu in the subarachnoid and perivascular spaces [53]. Under physiological conditions brain endothelial cells express the homeostatic chemokine CXCL12, which is released in a polarized fashion at the abluminal side allowing for the retention of CXCR4 expressing immune cells in the subarachnoid and perivascular spaces [76]. During neuroinflammation the atypical chemokine receptor 3 (ACKR3), a scavenger receptor for CXCL12, is upregulated on brain endothelial cells leading to reduced CXCL12 concentrations in the CSF space and allowing immune cells to sense inflammatory chemotactic cues from the CNS parenchyma [77]. CCL19 is the second homeostatic chemokine expressed by brain endothelial cells [78, 79]. As CCL19 is a soluble chemokine that does not bind to extracellular matrix, it may also regulate the retention of CCR7 expressing memory T‐cell subsets in the CSF space.

While a vast majority of studies have focused on investigating the migration of defined T‐cell subsets like Th1, Th17 or, to a lesser degree, CD8 T cells, there is not much knowledge on how the site of priming affects T‐cell migration into the CNS. Studies where EAE is induced by the adoptive transfer of T‐cell blasts have proposed the lung as a tissue licensing T cells to preferentially migrate to the CNS [80]. However, in vitro activated T‐cell blasts may not be imprinted by the trafficking programs as shown to occur during T‐cell priming in vivo. A recent study has used elegant in vivo labeling of antigen‐specific T cells to show that T cells activated in skin‐draining and gut‐draining lymph nodes can both home to the CNS, but show distinct trafficking properties [81]. In the context of neuroinflammation, it was shown that T cells primed in the gut‐draining lymph nodes infiltrated CNS white matter, while those primed in the skin‐draining lymph nodes express CXCR6 and can additionally infiltrate CNS gray matter. It will thus be important to further explore how site‐specific priming and activation of T cells will affect their CNS homing properties to improve our understanding if this affects their ability to cross specific brain barriers or to home to specific CNS regions.

## Role of the brain barriers in regulating afferent pathways of CNS immunity

Research from the past few decades has shown that the traditional concept that lymphatic vessels are either not involved in or had only a minor contribution for CNS drainage no longer holds true [82]. Classic experiments from over 100 years ago were interpreted as evidence for direct efflux pathways through arachnoid villi or granulations for CSF to dural venous sinus blood from the subarachnoid space [83, 84]. Despite the fact that these early investigators also demonstrated that tracers injected into the subarachnoid space reached lymphatic vessels, particularly in the nasal mucosa, connections to the lymphatic system were consistently minimized by later researchers [85, 86]. Studies in the 1980s and 1990s showed that the proportion of tracers injected into either the CSF or the parenchyma of the brain that could be recovered in cannulated cervical lymphatics reached as much as 50% in species such as rabbits and sheep [87, 88, 89]. Thus, a consensus of dual‐outflow pathways, both venous and lymphatic, was reached [87, 90, 91]. Recently, imaging studies using tracers in rodents have challenged the concept of any CSF drainage directly to blood [92]. Furthermore, studies have demonstrated that a potential efflux pathway to a network of dura mater lymphatic vessels may also be involved in the clearance of fluid, solutes and, likely also, cells from the CNS [93, 94]. Thus, it is now becoming evident that like almost all other tissues in the body, the drainage of fluid, antigens, and cells from the CNS occurs through routes leading to the lymphatic system. Yet, due to the lack of any *bona fide* lymphatic vessels within the brain and spinal cord parenchyma and the dual‐walled barrier anatomy of the CNS, it is clear that unique anatomical arrangements must exist for fluid and its components and especially for immune cells to reach the peripheral lymphatic system.

### Evidence from tracer studies for CSF efflux pathways to lymphatics

Several cranial nerves emerge through foramina at the base of the skull and routes along these nerves have been repeatedly implicated in CSF outflow since an initial report in 1869 by Schwalbe [82, 95]. Of these, the route along the olfactory nerves to the nasal submucosal lymphatics has been investigated most thoroughly (Fig. 1). The efflux pathway from the subarachnoid space to lymphatic vessels in the nasal tissue crosses the cribriform plate of the ethmoid bone. At this location, the bundles of olfactory nerves emerge from the olfactory bulb to extend through foramina in the cribriform plate to terminate their axons in the nasal epithelium lining the airways. The nerve bundles (*fila olfactoria*) are covered with sheaths of connective tissue that forms a fluid space that is considered to be continuous with the subarachnoid space. It is thought that CSF is driven into this space due to hydrostatic pressure gradients where it then gains access to the nasal mucosa interstitial tissue or perhaps even directly to lymphatic vessels within this tissue [90, 96]. The dense lymphatic network of the nasal mucosa then drains into the deep cervical lymph nodes. Solid evidence for the importance of this route in draining CSF has come from experiments that have sealed the cribriform plate in vivo and have demonstrated dramatic reductions in CSF‐administered tracer recovery outside the CNS, a rerouting of tracer distribution to the spine and, in some cases, even increases in intracranial pressure [97, 98, 99].

It still remains to be determined how intact the arachnoid or perineural layers of tissue are at this location and thus whether any barriers might exist for fluid, solutes, or cells to reach the lymphatic system. It has been proposed that barrier properties are no longer present once the nerves have penetrated through the cribriform plate, as the perineural layers become loosely adhered to the nerves with discontinuous junctions between cells [96, 100]. In recent studies in rodents, lymphatic vessels have been shown to completely cross through the cribriform plate to potentially access the subarachnoid space directly [94, 101–103]. In order for constituents of the CSF to directly drain into these vessels, the arachnoid barrier must be lacking in integrity somewhere at this anatomical location. Some evidence for this exists in developing rats and humans, as claudin‐11, a transmembrane tight junction molecule that was found to delineate the arachnoid barrier, was not found in the tissue immediately overlaying the cribriform plate [104, 105]. Thus, an open pathway may exist for CSF to reach the lymphatics within the nasal mucosa in a bulk flow manner, as shown by studies demonstrating different sized particles reaching draining lymph nodes or the systemic circulation with similar dynamics [92, 106].

Several studies have also demonstrated outflow of CSF tracers along with the perineural space that accompanies the optic nerves as they exit through their respective optic canal [92, 107–109]. Unlike the olfactory nerves, the optic nerve is covered by arachnoid mater cells and dura mater up to the point where the nerve enters the orbit, raising immediate yet to be answered questions as to how these meningeal layers are permissive for outflow and where exactly the lymphatic vessels are located that drain the fluid. Nonetheless, tracers have been found to reach the connective tissue around the orbit and efflux through lymphatic vessels that track along the facial vein to the superficial cervical (or mandibular) lymph nodes [92, 107]. There is also evidence of efflux along trigeminal and facial nerves [92, 110, 111]. Finally, tracers have been found to exit the skull at the jugular foramen, whether in a perineural fashion along the glossopharyngeal (cranial nerve IX), vagus (cranial nerve X), and accessory (cranial nerve XI) nerves or as an exit point for basal dural lymphatics [92, 112].

Compared to the cranial pathways, a lesser proportion of CSF is drained from the spinal column. Much focus has been on the regions at the dorsal nerve root, where the spinal nerves emerge from the CNS to the periphery and are accompanied by layers of the meninges in a similar manner to cranial nerves. These layers form what is known as the subarachnoid angle or cul‐de‐sac at the dorsal root ganglion [113]. Lymphatics within either the dura mater or epidural tissue are present at a high density at these locations in mice [114, 115]. Using near infrared (NIR) and magnetic resonance (MR) imaging in mice, we have determined that the major efflux pathways from the spine appear to be located at the lumbar and sacral nerve roots near the *filum terminale* [116], consistent with earlier studies [117, 118]. After efflux into the epidural tissue, lymphatic vessels transport the CSF tracers to sacral and deep iliac lymph nodes [116].

A network of lymphatic vessels in the dura mater was rediscovered in two studies published in 2015, triggering much research activity on this topic [93, 94]. These vessels had been previously described in many species, including humans, cats, dogs, and rats [101, 119–122]. Anatomically, these vessels exist as a small network of vessels in the dura mater lining the dorsal skull, closely associated with the dural venous sinuses (superior sagittal and transverse sinuses), with a more extensive network in the cerebellar ring and following the dural blood vessels at the base of the skull [93, 94]. These vessels appear to be different in morphology to lymphatic vessels in other organs, with very sparse valve or smooth muscle coverage throughout the network. With the exception of some basal dural lymphatics, they were also shown to have vascular endothelial‐cadherin junctions of the “zipper” phenotype, which are more associated with either collecting or developing lymphatic vessels, rather than the mature lymphatic capillaries usually responsible for fluid and cell entry [112]. In addition, they form late in the developmental process, at the postnatal stage in the mouse, and also appear to be quite sensitive to anti‐lymphangiogenic treatment [114].

Although these dural lymphatic vessels have been the subject of intensive research since their rediscovery, several questions remain regarding their role in efflux from the CNS. For one, their location has been firmly established to be within the dura mater layer of the meninges. This is significant, as the dural blood vessels lack the endothelial cell barrier properties that are found in the CNS parenchyma and, thus, this places the dural lymphatic vessels outside the arachnoid barrier isolating the CNS from the periphery [123]. Second, transgenic mice that were found to lack dural lymphatic vessels had neither detectable increase in fluid in the brain nor any elevation in intracranial pressure [94]. While experiments from some groups have been able to document uptake of tracers into these vessels after either CSF or parenchymal administration [93, 94, 112, 124], others have not [92]. There is also debate about the relative importance of the dural lymphatic vessels on the dorsal aspect of the skull versus those on the basal aspect [112]. Tracer studies have consistently shown a predominantly ventral flux of CSF [125, 126, 127] with only minimal transport of CSF to the dorsal aspect of the brain.

### Evidence from tracer studies for clearance of CNS interstitial fluid

**Fig. 2:**
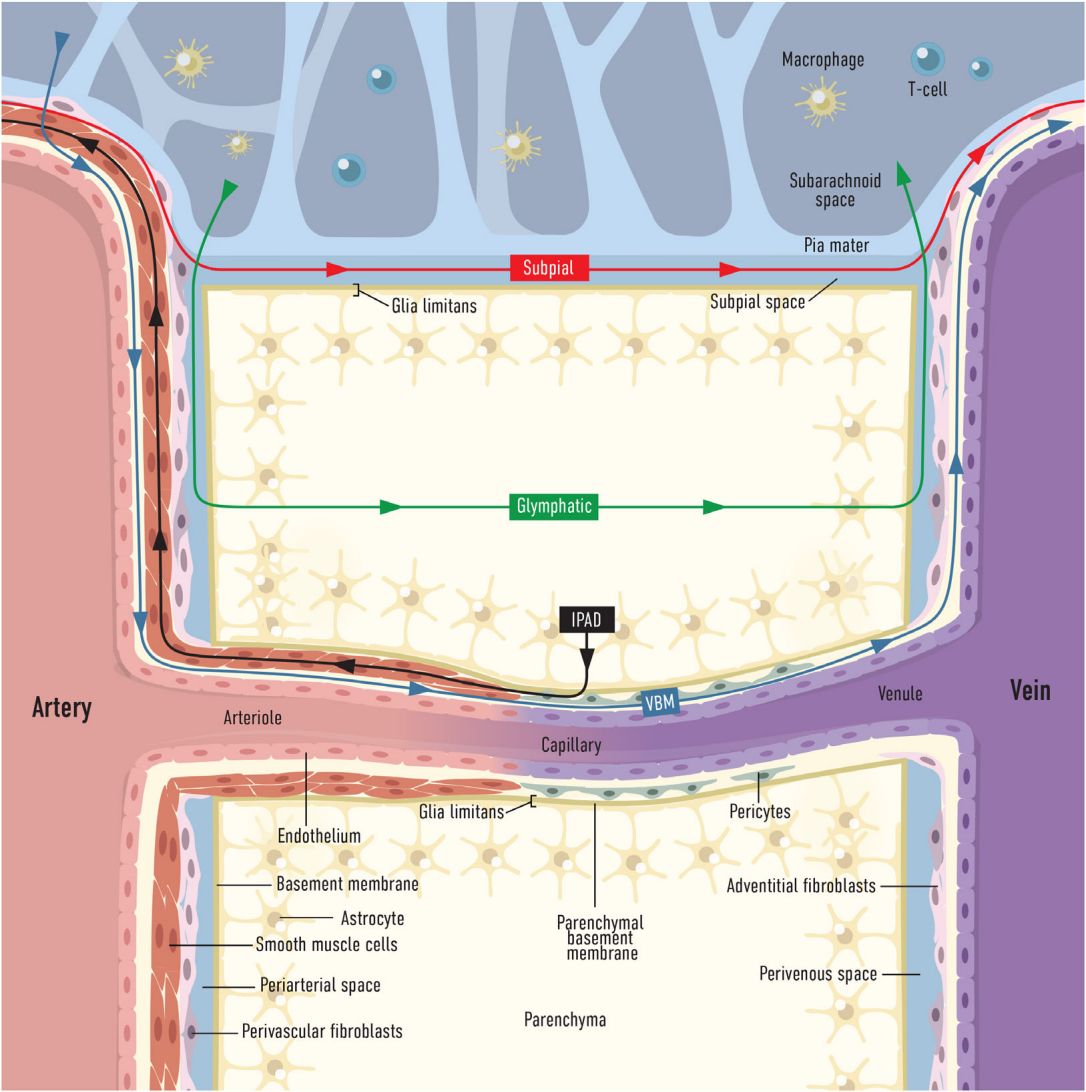
Proposed models of fluid flow and clearance in the mouse central nervous system (CNS). Interpretation of tracer studies in mice has led to the following models: (1) Glymphatic pathway (green): From the subarachnoid space, cerebrospinal fluid (CSF) enters a perivascular space located between the arterial wall and glia limitans along penetrating arterioles. CSF then flows through (or between) astrocyte end‐feet in an aquaporin‐4 (AQP4)‐dependent manner, to enter the brain parenchyma where it mixes with brain interstitial fluid (ISF) and waste metabolites. This fluid then, also through astrocyte‐mediated mechanisms, exits the brain interstitial space to reach a perivenous space and is directed either back to CSF or toward lymphatic outflow sites. (2) Vascular basement membrane pathway (VBM, blue): CSF enters through pia mater stomata (not shown) to subpial arteries on the brain surface. The fluid then gains access to vascular (endothelial, smooth muscle) basement membranes which provide low resistance fluid pathways for flow along the penetrating arterioles. At the capillary level this fluid pathway is defined by endothelial and parenchymal basement membranes that merge at this level. At the venule level this fluid pathway is proposed to continue within the endothelial basement membranes. In this model, mixing of CSF and brain ISF occurs predominantly via diffusion. (3) Intramural periarterial drainage pathway (IPAD, black): ISF and solutes from the brain parenchyma access the basement membranes at the brain capillaries and then drain along smooth muscle‐associated basement membranes around arterioles and arteries. This pathway is proposed to continue along the arteries as they breach the subarachnoid space to exit the skull, to reach extracranial lymphatics. (4) Subpial pathway (red): CSF has been shown to flow in a paravascular space (bordered by the vessel walls, the pia, and the glia limitans) on the surface of the brain in the direction of blood flow. This space appears to be created by a “tenting” of the pia over the surface arteries and veins creating a subpial space. At locations where arteries and veins are in close proximity or cross over each other, a transfer of fluid from paraarterial to paravenous spaces may occur.

Even more unsettled are the potential routes for the interstitial fluid (ISF) of the CNS parenchyma to clear to the periphery (Fig. 2). The traditional concept was that production of fluid occurs at the BBB with CSF acting as a “sink” for the fluid and solutes that need to be eliminated from the parenchyma [86]. This process was considered to be driven mostly by diffusive action through the parenchyma and then either through the ependyma into the ventricles or through the pia‐glia interface to reach the CSF in the subarachnoid space. Research from the group of Helen Cserr [128, 129] showed that parenchymal depots of different sized tracers clear at the same rate, indicating that bulk flow pathways must exist for clearance of the solutes. Based on the use of horseradish peroxidase, the efflux pathways were considered to be mostly along anatomically not precisely defined “perivascular spaces” of penetrating blood vessels to the subarachnoid space with lesser routes along fiber tracts of white matter and the subependyma [128]. While the mixing of interstitial solutes with the CSF appeared to be limited, at least at the cisterna magna sampling site, it was considered that the ISF still left the CNS via CSF efflux routes (e.g., nasal mucosa) [130]. However, another model has been proposed by other investigators to explain the lack of recovery of ISF‐injected tracers in the CSF [131, 132]. This model postulates that intramural basement membrane‐associated pathways starting at the level of capillary basement membranes and continuing in the vascular wall of arteries along the basement membranes of smooth muscle cells act as direct routes out of the brain, and that these “intramural periarterial drainage” (IPAD) spaces span the subarachnoid space and extend out of the skull to reach lymphatic vessels leading to the deep cervical lymph nodes [105, 133]. Although this continuous pathway from the brain to lymphatics has yet to be mapped, indirect evidence of perivascular edema and neurocognitive deficits after severing the cervical lymphatic connections has been observed in some species [134]. Additionally, the accumulation of amyloid, which occurs specifically around arteries, would also seem to provide support for such a clearance pathway [133].

Investigators have also proposed that CSF can enter the brain through anatomically not precisely defined paravascular/perivascular routes through a bulk flow mechanism. Traditional evidence for this has been based on postmortem assessments of tissue, in which tracer can be found at a perivascular location surrounding parenchymal blood vessels, even with short periods of time between the time of injection and death of the animal [135, 136, 137]. However, it has been shown that at death, a massive and rapid influx of tracers into perivascular spaces of CNS penetrating vessels can occur, which will complicate the conclusions that can be made from analysis of postmortem specimens [126]. Stronger evidence would appear to exist from in vivo experiments, using epifluorescence or two‐photon intravital microscopy measurements of particle flow at the cortical surface of the brain in rodents. With two‐photon intravital microscopy measurements through a cranial window, Iliff et al. demonstrated that tracers appeared to enter the perivascular space of penetrating arterioles soon after injection into the cisterna magna [138]. At later time points, the tracers appeared in paravascular spaces of veins on the brain surface. In mice lacking aquaporin‐4 (AQP4), a water channel highly polarized at the end‐feet of astrocytes of the glia limitans, less influx was observed. These observations led to the proposal of the existence of a “glymphatic system,” in which CSF would enter in a convective manner along para/periarterial routes, flow through the AQP4 water channels across astrocyte end‐feet to flush through the brain parenchyma while mixing with ISF, and then exit in a similar manner at peri‐/paravascular routes along veins [138]. At this point, the fluid has been speculated (but not yet shown) to gain access to the lymphatic system [139]. In subsequent studies, this group has observed a paravascular flow of particles in the direction of blood flow along arteries on the surface of the brain, possibly in a subpial location, a flow that is assumed to continue to perivascular spaces around the arteries as they penetrate into the brain parenchyma [140]. However, one must note that tracers may directly reach paravenous spaces on the surface of the brain without apparent influx into the brain [126], thus the mechanisms controlling fluid flow in paravascular spaces around blood vessels on the surface of the brain may be distinct from those that may exist for perivascular routes within the parenchyma.

At this point, it is important to note that experimental and modeling studies have raised many issues regarding the existence of the glymphatic system [126, 141–143]. A shortcoming of the glymphatic concept is that it lacks integration of the existing cellular and acellular brain barriers. The proposed mixing of CSF with ISF by the glymphatic concept is also difficult to reconcile with the maintenance of a homeostatic environment of the CNS parenchyma that is essential for proper neuronal function. Furthermore, the glymphatic concept does not provide a satisfying answer on how the proposed efficient “waste clearance” of the CNS parenchyma via the CSF would allow to maintain immune privilege of the CNS parenchyma while directing immune surveillance to the CSF‐filled CNS border compartments. Taking these considerations into account, another model has been proposed in which a directed circulation of CSF occurs along vascular basement membranes that could provide a potential low resitance route for fluid and solute flow within the perivascular compartment at all levels of the vascular tree [125, 142]. In this model, the exchange of ISF in the parenchyma and the CSF in the perivascular space would occur primarily through diffusion across the glia limitans (Fig. 2).

Of interest is that “glymphatic clearance” of the CNS is more active during sleep [144] while lymphatic drainage in the periphery depends on muscular activity and is thus higher during awake periods [145]. Combined with the observation that immune cell trafficking and function shows circadian clock driven rhythmicity [146], it will be important to bring these observations into an agreement to improve our understanding of CNS immunity.

## Afferent pathways during CNS immune responses

Many fundamental questions remain about how soluble antigens or APCs utilize potential efflux pathways for initiation of an adaptive immune response or the maintenance of peripheral tolerance to CNS autoantigens. In the periphery, antigen‐specific activation of lymphocytes may occur within the affected organ itself, along the pathways of antigen drainage or within the draining lymphoid tissue. Almost all tissues in the body contain strategically placed APCs capable of presenting antigens by MHC class I or II molecules to CD8^+^ and CD4^+^ T cells, respectively. As outlined above, initiation of immune responses within the CNS parenchyma itself has not been readily observed. Instead, soluble antigens may be drained along the pathways that have been proposed by the aforementioned tracer studies for uptake and presentation within the CNS‐draining lymph nodes or, alternatively, CNS border‐associated APCs take up and present antigens locally or may potentially traffic from the CSF spaces to the lymph nodes.

Pioneering work by the group of Helen Cserr and Paul Knopf in the late 1980s and early 1990s, investigated systemic humoral immune responses in rats using CSF injections through an indwelling cannula of different foreign antigens, such as human serum albumin (HSA) or chicken ovalbumin, or autoantigens such as myelin basic protein (MBP). Low volume injections of either HSA or ovalbumin into the CSF resulted in an antigen‐specific IgG serum antibody response with antibody‐secreting cells identified in both superficial and deep cervical lymph nodes and the spleen [147, 148]. Interestingly, injections of HSA into CSF were more immunogenic than when injected into extracerebral sites, such as the peritoneal cavity or muscle. Lymphatic efflux to cervical lymph nodes was essential for these responses as 10‐fold less antigen‐specific antibody could be detected in serum if cervical lymph nodes were surgically removed prior to the antigen injection. It will be interesting to explore if this efficient CSF‐raised CNS‐specific humoral immune response observed in these early studies receives a contribution of the recently identified “private CNS source of B cells” residing in the dura mater [39, 45]. These dural resident B cells have been proposed to maintain CNS immune quiescence [39]. In accordance with this recent concept, the early work in the Cserr laboratory already showed that an infusion of the autoantigen MBP into the CSF of rats before induction of EAE by subcutaneous immunization with MBP in adjuvants suppressed clinical symptoms in this rat EAE model [149]. Interestingly, MPB infusion into the CSF was more effective at suppressing clinical signs of EAE when compared to systemic injection of MPB [149], suggesting that appearance of soluble protein antigens in the CSF rather leads to tolerizing immune responses. Since this time, several other studies have demonstrated the importance of an intact communication with CNS‐draining lymph nodes during the progression of EAE. For example, removal of the cervical lymph nodes reduced the size of the cortical lesions in cryolesion‐enhanced EAE in rats [150] and attenuated the symptoms of spontaneous EAE in a transgenic mouse model [151]. In an elegant study, excision of superficial and deep cervical lymph nodes and as well as the spine‐draining lumbar lymph nodes limited the severity of relapses in an actively induced relapsing‐remitting EAE mouse model [152]. In a more recent study, Louveau et al. used a cisterna magna‐injected Visudyne compound known to generate destructive reactive oxygen species through activation by light shown through the skull to ablate specifically the dorsal lymphatic vessel structures. In an EAE model, this led to delayed onset of the disease with reduced clinical symptoms [124]. Surprisingly, neuroinflammation does not appear to lead to an expansion of the dural lymphatic vessels [103, 124], despite their apparent sensitivity to lymphangiogenic compounds.

While it is generally now accepted that border‐associated macrophages such as perivascular macrophages [153, 154] and others are the principle APCs within the CNS [155], there is yet no robust evidence that these resident cells traffic to draining lymph nodes. Several studies have now provided evidence for the presence of bona fide DCs in CNS subarachnoid and perivascular locations during homeostatic conditions [155, 156]. Immune cells, including DCs, have been shown to egress through the cribriform plate to the nasal mucosa tissue, although these studies have typically been able to identify only a few cells in each case [103, 124, 157, 158]. If initial lymphatic vessels found on the CNS‐facing side of the cribriform plate truly extend into the subarachnoid space, this may allow for unfettered APC access to the lymphatic system. Lymphatic vessels on the CNS side of the cribriform plate express CCL21 [103], the major chemokine implicated in immune cell migration into initial lymphatic vessels through interactions with CCR7 [159, 160]. Indeed, cells lacking CCR7 are retained to a greater degree in the CNS than those that express this receptor [124, 161]. Further research is necessary to elucidate whether APC migration from the CNS plays a significant role in disease. While APCs containing myelin and axonal antigens have been found within cervical lymph nodes from patients with MS, and mouse and primate models of EAE [162], it is not clear whether these antigens were transported within these cells or were rather taken up in soluble form within the afferent lymph arriving at the subcapsular sinus.

Recently, the choroid plexus and the dura mater have been implicated as important neuroimmune interfaces. However, a key question that remains is how these immune cell ramparts in the choroid plexus stroma and the dura mater could potentially communicate with the CNS border compartments or the CNS parenchyma proper that are separated from these sites by one or even two brain barriers. The choroid plexus has no known lymphatic vessel network and, thus, would not be an expected site for the initiation of a classical systemic adaptive immune response. Yet, one study has proposed that T cells injected into the lateral ventricle can migrate across the choroid plexus epithelium and undergo activation by APCs within the choroid plexus stroma [163]. It will remain to be shown if the choroid plexus stroma provides a unique immunological niche that ensures CNS immune surveillance by allowing for two‐way trafficking of immune cells across the BCSFB and thus integration of signals from the periphery and the CNS.

It is also intriguing that immune cells in the dura mater are not found to be evenly distributed but rather observed to accumulate close to the dural venous sinuses [39, 40]. This precise site was previously identified as a neuroimmune interface where CSF‐derived antigens can accumulate and lead to local T‐cell activation [74]. As at precisely these locations veins from the CNS parenchyma and subarachnoid space egress into the dural sinus, it is tempting to speculate that molecules from the CNS parenchyma or subarachnoid space may reach the dura mater by moving along the walls of blood vessels that bridge the glia limitans and the arachnoid barrier. One could also speculate that this site allows for the trafficking of immune cells between the dura mater and the subarachnoid space potentially circumventing the arachnoid barrier.

In addition, the CSF‐draining perineural spaces have yet to be closely examined as potential sites of immunosurveillance. While peripheral nerve myeloid‐derived cells have recently been identified [164, 165], it is yet to be determined whether APCs and lymphocytes can traffic to and from these sites from the periphery.

## Conclusions and outlook

Our concept where the brain barriers play a central role in maintaining CNS immune privilege and immune surveillance raises the question of how these barriers form during embryonic development allowing for the establishment of CNS immune privilege. Protein concentrations in fetal CSF are much higher when compared to the adult. While some authors suggested that this is due to the embryonic immaturity of the BBB and BCSFB, others have shown that the high CSF protein concentration is rather a result of transcellular transfer of plasma proteins across the embryonic choroid plexus epithelial cells (summarized in [166]). Combined with slow CSF protein turnover and an ependymal CSF‐brain barrier, which is only a barrier between the CSF and CNS parenchyma during embryonic development, this would provide an alternative explanation for the high CSF protein concentrations detected during development. These observations highlight that localization of the barriers dividing CNS compartments with respect to their accessibility for blood‐derived proteins may change during development. In addition, characteristics of a given barrier may change during development. This is true for the BBB across which access of immune cells to the CNS changes during development. During early postnatal development, the BBB still allows for CNS access by circulating naïve T cells, which is no longer observed in the adult [167, 168]. By allowing CNS access of naïve T cells early during development the BBB may play an active role in establishing peripheral self‐tolerance to CNS‐sequestered antigens [169]. It is intriguing to note that in contrast to most lymphatic vessels, which mature during embryonic development, the dural lymphatics only develop postnatally [114], suggesting that early availability of this drainage pathway would interfere with the development of CNS immunity.

Communication between the brain barriers with a potential impact in adapting barrier functions has also been observed in the adult where in case of neuroinflammation when BBB properties fail, induction of tight junctions between astrocyte end‐feet was observed suggesting establishment of a novel glial BBB for immune cell and immune‐mediator entry into the CNS [170].

An increasing number of studies have provided evidence for a role of BBB alterations established during development in causing psychotic disorders like schizophrenia. These observations converge with increasing evidence for a role of immune dysfunction in these diseases [171] pointing to an important role of the brain barriers in cognitive functions and CNS immunity. In fact, studies pioneered by the laboratory of Michal Schwartz have provided evidence that CNS‐specific adaptive immunity is required for brain plasticity and neuronal function [172]. This “protective autoimmunity” was shown to be mediated by T cells from the CNS borders.

Further elucidation of the role of the individual brain barriers and how they control the entry and exit of immune cells and soluble immune mediators in the CNS may lead to new therapies for patients with neuroinflammatory disorders focused on limiting the severity of clinical disability or preventing secondary progression. In this context, the development of imaging modalities allowing determination of the functional and/or inflammatory status of the different brain barriers by magnetic resonance imaging or positron emission tomography imaging in humans would significantly advance diagnosis and treatment of neuroinflammatory disorders. To this end, studies exploring the molecular signatures of the brain barriers during health and neuroinflammation will set the stage for future developments of barrier‐specific imaging modalities. In this context, the recent high profile studies that have suggested possible roles for immune cells at the choroid plexus and the dura mater will spark this research on both the preclinical and clinical levels to find potential new targets for diagnosis and treatment at these CNS border tissues.

A better understanding of the compartmental nature of the CNS may also improve drug delivery for treatment of neurological conditions. It remains a significant challenge for systemically administered compounds to reach target cells in the CNS parenchyma as they must not only breach the BBB or BCSFB but also the additional barriers at the pia mater and/or glia limitans. Alternative strategies may emerge utilizing intrathecal or intranasal administration to target immune cell aggregates within the CSF compartment or to modulate lymphatic efflux.

In conclusion, the orchestrated function of the different endothelial, epithelial, glial, and fibroblast‐derived brain barriers in zoning the CNS has not received sufficient attention. Integrating our growing mechanistic understanding of the function of these brain barriers in zoning the CNS into compartments with different accessibility to immune cells and immune mediators will be of fundamental importance to understand how these neuroimmunological interfaces shape CNS immunity and neuronal activity and thus ultimately CNS health and disease.

## Conflict of interest

The authors declare no conflict of interest.
